# Evolution of Electroconvulsive Therapy during the COVID-19 pandemic in the Regional ECT Unit of the Region of Murcia

**DOI:** 10.1192/j.eurpsy.2022.1333

**Published:** 2022-09-01

**Authors:** M.A. Cutillas Fernández, M.L. Medina Garrido, M. López Villaescusa, E. Bermudez Larrosa, C. Martínez Milán, E. Aparicio Castro

**Affiliations:** 1 Hospital Morales Meseguer, Psiquiatría, Murcia, Spain; 2 Hospital Psiquiátrico Román Alberca, Unidad Regional De Tec, Murcia, Spain

**Keywords:** Maintenance ECT, Covid-19, Electroconvulsive therapy

## Abstract

**Introduction:**

Covid-19 was declared a global pandemic by the WHO on 11 March 2020. From the beginning, the pandemic posed a challenge to the different health systems around the world, which were forced to prioritise and distribute their resources as efficiently as possible. During the period between 11 March 2020 and 28 April 2021, the Regional ECT Unit of the Region of Murcia remained closed.

**Objectives:**

- Determine the clinical status of patients on maintenance ECT in the Regional ECT Unit during the Covid-19 pandemic.

- Prioritise resumption of treatment in those who were clinically decompensated or at risk

- Understand the consequences of discontinuation of maintenance ECT for these patients.

**Methods:**

A longitudinal descriptive study was conducted during the month of May 2020.

**Results:**

Thirty-seven patients were contacted by telephone. On the first call, a total of 15 patients were unstable or at risk of decompensation.

Prior to the second call, CT was administered preferentially to a total of 8 patients and programmed to 2. On the second call, a total of 11 patients were at risk of decompensation.

**Conclusions:**

The closure of the Regional ECT Unit had negative consequences for patients undergoing maintenance treatment. Electroconvulsive therapy is an essential part of the treatment of psychiatric patients both in acute episodes and in relapse prevention.

**Disclosure:**

No significant relationships.

